# Supplementary material for: Discovery and Validation of Survival-Specific Genes in Papillary Renal Cell Carcinoma Using a Customized Next-Generation Sequencing Gene Panel
Source: Cancers (Basel). 2024 May 25;16(11):2006. doi: 10.3390/cancers16112006 (PMC11171119; doi:10.3390/cancers16112006)
Supplement: Supplementary file 1 [file cancers-16-02006-s001.zip › Supplementary file_Table.pptx]

## Slide 1
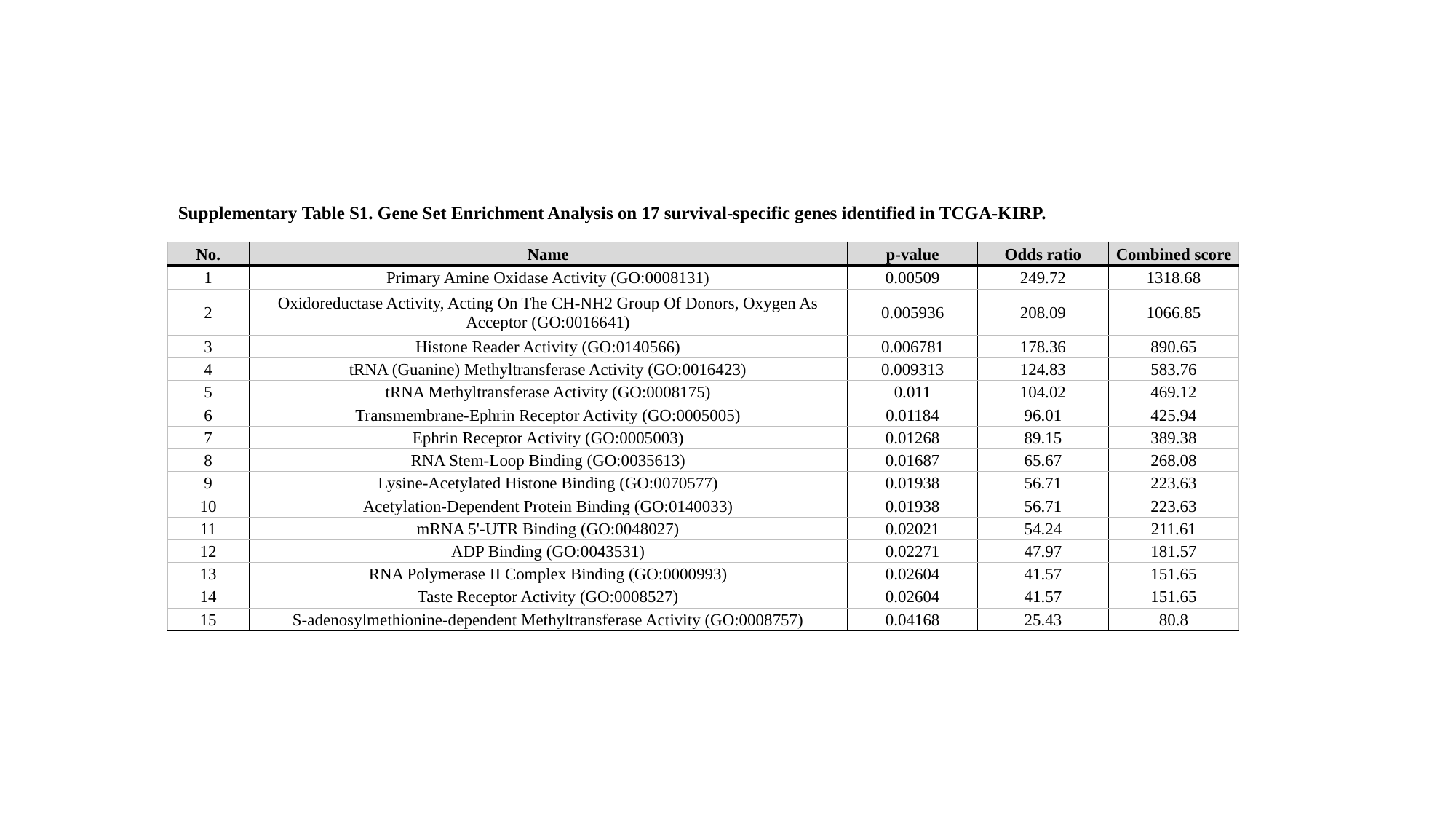

Supplementary Table S1. Gene Set Enrichment Analysis on 17 survival-specific genes identified in TCGA-KIRP.
| No. | Name | p-value | Odds ratio | Combined score |
| --- | --- | --- | --- | --- |
| 1 | Primary Amine Oxidase Activity (GO:0008131) | 0.00509 | 249.72 | 1318.68 |
| 2 | Oxidoreductase Activity, Acting On The CH-NH2 Group Of Donors, Oxygen As Acceptor (GO:0016641) | 0.005936 | 208.09 | 1066.85 |
| 3 | Histone Reader Activity (GO:0140566) | 0.006781 | 178.36 | 890.65 |
| 4 | tRNA (Guanine) Methyltransferase Activity (GO:0016423) | 0.009313 | 124.83 | 583.76 |
| 5 | tRNA Methyltransferase Activity (GO:0008175) | 0.011 | 104.02 | 469.12 |
| 6 | Transmembrane-Ephrin Receptor Activity (GO:0005005) | 0.01184 | 96.01 | 425.94 |
| 7 | Ephrin Receptor Activity (GO:0005003) | 0.01268 | 89.15 | 389.38 |
| 8 | RNA Stem-Loop Binding (GO:0035613) | 0.01687 | 65.67 | 268.08 |
| 9 | Lysine-Acetylated Histone Binding (GO:0070577) | 0.01938 | 56.71 | 223.63 |
| 10 | Acetylation-Dependent Protein Binding (GO:0140033) | 0.01938 | 56.71 | 223.63 |
| 11 | mRNA 5'-UTR Binding (GO:0048027) | 0.02021 | 54.24 | 211.61 |
| 12 | ADP Binding (GO:0043531) | 0.02271 | 47.97 | 181.57 |
| 13 | RNA Polymerase II Complex Binding (GO:0000993) | 0.02604 | 41.57 | 151.65 |
| 14 | Taste Receptor Activity (GO:0008527) | 0.02604 | 41.57 | 151.65 |
| 15 | S-adenosylmethionine-dependent Methyltransferase Activity (GO:0008757) | 0.04168 | 25.43 | 80.8 |

## Slide 2
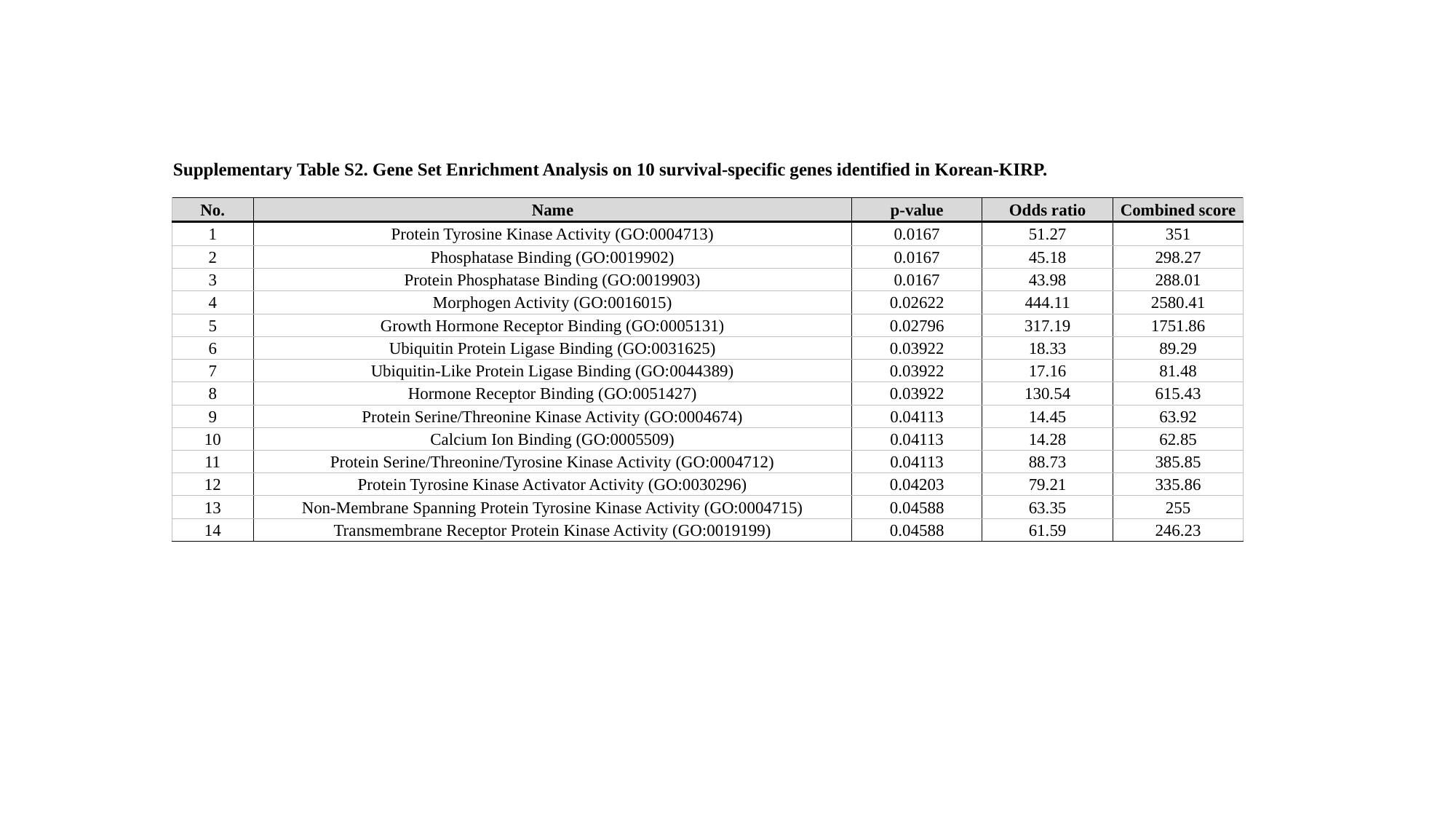

Supplementary Table S2. Gene Set Enrichment Analysis on 10 survival-specific genes identified in Korean-KIRP.
| No. | Name | p-value | Odds ratio | Combined score |
| --- | --- | --- | --- | --- |
| 1 | Protein Tyrosine Kinase Activity (GO:0004713) | 0.0167 | 51.27 | 351 |
| 2 | Phosphatase Binding (GO:0019902) | 0.0167 | 45.18 | 298.27 |
| 3 | Protein Phosphatase Binding (GO:0019903) | 0.0167 | 43.98 | 288.01 |
| 4 | Morphogen Activity (GO:0016015) | 0.02622 | 444.11 | 2580.41 |
| 5 | Growth Hormone Receptor Binding (GO:0005131) | 0.02796 | 317.19 | 1751.86 |
| 6 | Ubiquitin Protein Ligase Binding (GO:0031625) | 0.03922 | 18.33 | 89.29 |
| 7 | Ubiquitin-Like Protein Ligase Binding (GO:0044389) | 0.03922 | 17.16 | 81.48 |
| 8 | Hormone Receptor Binding (GO:0051427) | 0.03922 | 130.54 | 615.43 |
| 9 | Protein Serine/Threonine Kinase Activity (GO:0004674) | 0.04113 | 14.45 | 63.92 |
| 10 | Calcium Ion Binding (GO:0005509) | 0.04113 | 14.28 | 62.85 |
| 11 | Protein Serine/Threonine/Tyrosine Kinase Activity (GO:0004712) | 0.04113 | 88.73 | 385.85 |
| 12 | Protein Tyrosine Kinase Activator Activity (GO:0030296) | 0.04203 | 79.21 | 335.86 |
| 13 | Non-Membrane Spanning Protein Tyrosine Kinase Activity (GO:0004715) | 0.04588 | 63.35 | 255 |
| 14 | Transmembrane Receptor Protein Kinase Activity (GO:0019199) | 0.04588 | 61.59 | 246.23 |

## Slide 3
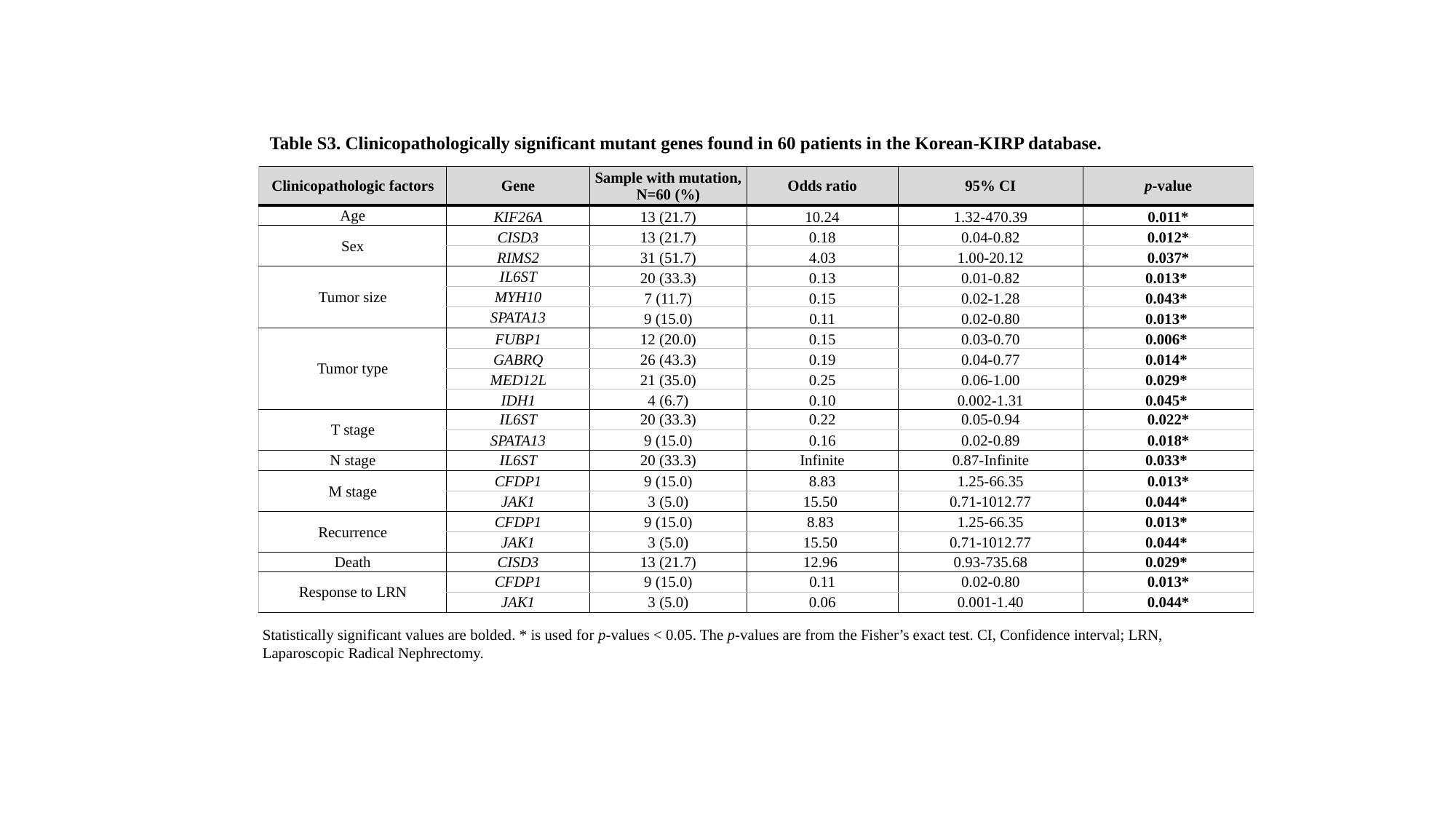

Table S3. Clinicopathologically significant mutant genes found in 60 patients in the Korean-KIRP database.
| Clinicopathologic factors | Gene | Sample with mutation, N=60 (%) | Odds ratio | 95% CI | p-value |
| --- | --- | --- | --- | --- | --- |
| Age | KIF26A | 13 (21.7) | 10.24 | 1.32-470.39 | 0.011\* |
| Sex | CISD3 | 13 (21.7) | 0.18 | 0.04-0.82 | 0.012\* |
| | RIMS2 | 31 (51.7) | 4.03 | 1.00-20.12 | 0.037\* |
| Tumor size | IL6ST | 20 (33.3) | 0.13 | 0.01-0.82 | 0.013\* |
| | MYH10 | 7 (11.7) | 0.15 | 0.02-1.28 | 0.043\* |
| | SPATA13 | 9 (15.0) | 0.11 | 0.02-0.80 | 0.013\* |
| Tumor type | FUBP1 | 12 (20.0) | 0.15 | 0.03-0.70 | 0.006\* |
| | GABRQ | 26 (43.3) | 0.19 | 0.04-0.77 | 0.014\* |
| | MED12L | 21 (35.0) | 0.25 | 0.06-1.00 | 0.029\* |
| | IDH1 | 4 (6.7) | 0.10 | 0.002-1.31 | 0.045\* |
| T stage | IL6ST | 20 (33.3) | 0.22 | 0.05-0.94 | 0.022\* |
| | SPATA13 | 9 (15.0) | 0.16 | 0.02-0.89 | 0.018\* |
| N stage | IL6ST | 20 (33.3) | Infinite | 0.87-Infinite | 0.033\* |
| M stage | CFDP1 | 9 (15.0) | 8.83 | 1.25-66.35 | 0.013\* |
| | JAK1 | 3 (5.0) | 15.50 | 0.71-1012.77 | 0.044\* |
| Recurrence | CFDP1 | 9 (15.0) | 8.83 | 1.25-66.35 | 0.013\* |
| | JAK1 | 3 (5.0) | 15.50 | 0.71-1012.77 | 0.044\* |
| Death | CISD3 | 13 (21.7) | 12.96 | 0.93-735.68 | 0.029\* |
| Response to LRN | CFDP1 | 9 (15.0) | 0.11 | 0.02-0.80 | 0.013\* |
| | JAK1 | 3 (5.0) | 0.06 | 0.001-1.40 | 0.044\* |
Statistically significant values are bolded. * is used for p-values < 0.05. The p-values are from the Fisher’s exact test. CI, Confidence interval; LRN, Laparoscopic Radical Nephrectomy.

## Slide 4
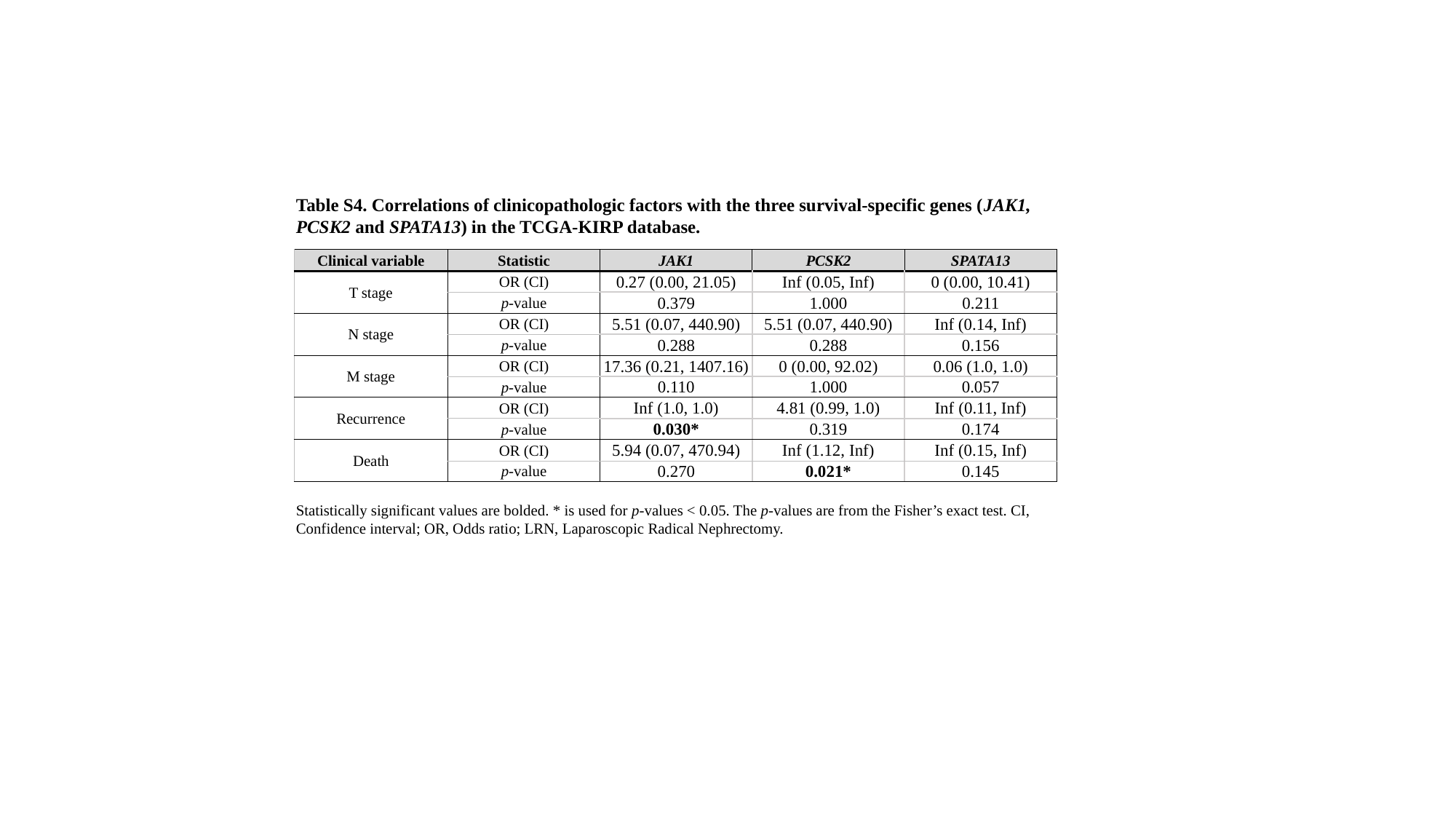

Table S4. Correlations of clinicopathologic factors with the three survival-specific genes (JAK1, PCSK2 and SPATA13) in the TCGA-KIRP database.
| Clinical variable | Statistic | JAK1 | PCSK2 | SPATA13 |
| --- | --- | --- | --- | --- |
| T stage | OR (CI) | 0.27 (0.00, 21.05) | Inf (0.05, Inf) | 0 (0.00, 10.41) |
| | p-value | 0.379 | 1.000 | 0.211 |
| N stage | OR (CI) | 5.51 (0.07, 440.90) | 5.51 (0.07, 440.90) | Inf (0.14, Inf) |
| | p-value | 0.288 | 0.288 | 0.156 |
| M stage | OR (CI) | 17.36 (0.21, 1407.16) | 0 (0.00, 92.02) | 0.06 (1.0, 1.0) |
| | p-value | 0.110 | 1.000 | 0.057 |
| Recurrence | OR (CI) | Inf (1.0, 1.0) | 4.81 (0.99, 1.0) | Inf (0.11, Inf) |
| | p-value | 0.030\* | 0.319 | 0.174 |
| Death | OR (CI) | 5.94 (0.07, 470.94) | Inf (1.12, Inf) | Inf (0.15, Inf) |
| | p-value | 0.270 | 0.021\* | 0.145 |
Statistically significant values are bolded. * is used for p-values < 0.05. The p-values are from the Fisher’s exact test. CI, Confidence interval; OR, Odds ratio; LRN, Laparoscopic Radical Nephrectomy.
